# How insurance affects altruistic provision in threshold public goods games

**DOI:** 10.1038/srep09098

**Published:** 2015-03-13

**Authors:** Jianlei Zhang, Chunyan Zhang, Ming Cao

**Affiliations:** 1 Theoretical Biology Group, Groningen Institute for Evolutionary Life Sciences, University of Groningen, The Netherlands; 2 Network Analysis and Control Group, Engineering and Technology Institute Groningen, University of Groningen, The Netherlands; 3 College of Computer and Control Engineering, Nankai University, China

## Abstract

The occurrence and maintenance of cooperative behaviors in public goods systems have attracted great research attention across multiple disciplines. A threshold public goods game requires a minimum amount of contributions to be collected from a group of individuals for provision to occur. Here we extend the common binary-strategy combination of cooperation and defection by adding a third strategy, called insured cooperation, which corresponds to buying an insurance covering the potential loss resulted from the unsuccessful public goods game. Particularly, only the contributing agents can opt to be insured, which is an effort decreasing the amount of the potential loss occurring. Theoretical computations suggest that when agents face the potential aggregate risk in threshold public goods games, more contributions occur with increasing compensation from insurance. Moreover, permitting the adoption of insurance significantly enhances individual contributions and facilitates provision, especially when the required threshold is high. This work also relates the strategy competition outcomes to different allocation rules once the resulted contributions exceed the threshold point in populations nested within a dilemma.

The origin and stability of cooperation is a hot subject in social and behavioural sciences^1^,^2^. A complicated conundrum exists as defectors have an advantage over cooperators, whenever cooperation is costly and consequently, defection pays off. Therefore social dilemmas are situations in which the optimal decision of an individual contrasts with the optimal decision for the group^3^,^4^,^5^. In the investigation of this plight the most prevailing framework is game theory together with its extensions involving evolutionary context^6^,^7^,^8^,^9^,^10^.

Throughout evolution, crucial human activities like hunting for food, conserving common forestry or fisheries resources, and warfare, constitute public goods. In situations like these, each group member gains benefits from the goods, including those who pay no cost of providing the goods. This arouses the question of why characters regularly participate in costly cooperative activities like warfare and risky hunting. Perhaps one of the most frequently used multiple-agent-two-strategy models to describe the confusion of how cooperation arises is the public goods game (PGG)^11^,^12^,^13^,^14^,^15^. It focuses on the gains arising in multi-person interactive decision situations when probably a part of the population decide to cooperate^16^,^17^,^18^,^19^.

Quite a few solutions or mechanisms have been put forward to explain the perplexing problem of cooperation evolution. The kin selection theory focuses on cooperation among individuals that are genetically related, whereas theory of direct reciprocity emphasizes the selfish incentives for cooperation in bilateral long-term interactions^20^,^21^,^22^. The theories of indirect reciprocity and signalling indicate how cooperation in larger groups can emerge when cooperators can build a reputation^23^,^24^. Besides, punishment also plays a crucial role in the resolution of cooperative dilemma^25^,^26^,^27^,^28^,^29^. The integration of the microscopic patterns of interactions among the individuals composing a large population into the evolutionary setting, affords a way out for cooperators to survive in paradigmatic scenarios. A common framework is that each node in a graph carries one player and, edges determine who plays with whom^30^,^31^,^32^,^33^.

Although the public goods game is deemed as one of the most common games in the study of the cooperation evolution, there are still some social dilemmas for which a different game would be a more appropriate model. In many cases of a collective action, the achieving of the group goal depends on the amount of common goods contributions. It is a common observation that many public goods contributed by collective actions are provided if contributions reach or exceed the required threshold of contributions; otherwise, no goods is provided^34^,^35^,^36^. Thus, a threshold public goods game requires a minimum amount of contributions to be raised from a group of individuals for provision to occur^37^,^38^,^39^. Researchers have examined how several factors, including incomplete information and identifiability of individual contributions, inhibit or foster successful public goods provision^40^,^41^,^42^,^43^,^44^,^45^,^46^,^47^.

Our previous work^48^ has introduced insurance against punishment and studied the roles of speculation adopted by defectors in public goods systems. Along this line, our aim here is to devise a scenario of evolutionary competition between three competing strategies, and study the roles of insurance for cooperators in the promotion of public cooperation. We are interested in the capacity of agents to contribute and produce the public goods when they are confronted with ambiguous risks or losses, meanwhile, facing the choice of being insured. In this threshold public goods model, agents can buy an insurance that sequentially covers the potential loss. We consider these aspects in an insurance deal, since the premium should not only be high enough to compensate the insurer for bearing the individual's risk, it should at the same time be low enough so that an individual is willing to insure her risk for this premium. Besides, if the threshold is not reached, contributions are not returned to the providers.

We add the insured cooperation as the third strategy to extend the individual strategy profiles originally consisting of cooperation and defection. These sets of hypotheses are generated from the motivation of our designing insurance in threshold public goods game. In the first place, everyday experience tells us that agents differ in personal features, such as the often-observed different economic status, or consciousness and demand for insurance in real world. When facing some potential loss, players may show heterogeneity in risk preferences. There is one more paramount point, cooperators will lose all their contributions when the group contribution falls short of the threshold. Intuitively, it is reasonable that the purchasers of insurance are cooperators since they are the altruistic contributors of the public goods activities and bear the risk of losing all their contributions. Therefore it is fair that they are provided the option of transferring their future loss to some insurance policy. In doing so, they could get some (part or full) compensation for their altruistic behaviors. In other words, the proposed insurance mechanism is provided as a means for encouraging those contributors and an effort deceasing the size of any loss occurring. Conversely, defectors can rest easy with no contribution for the generation of common goods. In this sense, it is meaningful to provide insurance choice only for contributors to avoid or decrease the unfavorable loss in this game setting. Finally, it is plausible that the insurance provider may be a profit management, and it will prefer cooperators over defectors as the object of insurance. The reason is that success accomplishment of public goods will help the insurance company save more benefits, otherwise it has to cover the loss for the insurers. Especially, the success provision of public goods is closely related to the number of cooperators. In this new framework, the two-strategy public goods game can be convincingly reframed as a cooperative dilemma among cooperators, insured cooperators and free riders.

The rest of the paper proceeds as follows. In section 2 we describe the threshold public goods games with three strategies in the static context. Next, we present and discuss the main dynamic outcomes of the system, whereas conclusive remarks are given in the final section.

## The model

In a typical threshold public good game (TPGG), each player in a group receives an endowment and individually decides how much of it to be contributed to a public goods system. If the group contribution exceeds a certain threshold, then the public goods is successfully provided and each player receives an equal reward, irrespective of her strategy. If the threshold is not reached, contributions are not returned to the players. Rational players intend to selfishly free ride on others' contributions, as contributors always benefit others at a cost to their benefits. Therefore, this rationale leads to social dilemmas and the predictable abandonment of the public goods.

To illustrate, suppose that in a finite population of size *N* (*N* > 1), individuals are provided with identical endowment *c*, and each must privately decide how much (between all and none here) of her endowment to contribute. After multiplying the accumulated contributions by *r*, each individual receives an identical benefit, if the required threshold T* is reached by the group as a whole. Note that *rc* < T* < *rcN* so that it is impossible for the threshold to be reached based solely upon the contribution of one player, but it is possible for it to be attained based upon the contributions of more than one player^19^.

As mentioned, when facing with potential loss, some cooperators prefer buying an insurance covering the possible loss and we call them the insured cooperators. Other cooperators may disregard this insurance and readily bear the potential loss, and they will be referred to as (common) cooperators. For the public goods game played by *N* players, both of the insured cooperators and common cooperators are contributors and their numbers are denoted by *N_i_* (insured cooperators) and *N_c_* (cooperators) respectively. Thus the population is composed of *N_i_* + *N_c_* contributors and *N_d_* free-riders.

Next, if the threshold is already achieved, how to define the payoff function of the participants gained from more contributions and provision is a crucial step. For the sake of generality, herein we consider two types of payoff functions that are plausible and conform to real situations for the study of cooperation, described in the following two scenarios respectively.

Scenario I: If the group contribution exceeds the required threshold, all the participants will henceforth share the fixed return T*/*N* from the accomplished public goods game. And that is, contributions above the threshold point of provision are wasted. There exists a set of living examples conforming to this model setting, such as voting for building a public garden or dam. The neighborhood residents are asked to individually fill in a questionnaire, or vote, or petition the government to get the project approved. For example, whether the public project will be approved and built, depends on the amount of supporters and the required minimum numbers needed for successful action. The residents might not know how many signatures are needed to get the project built. In the example above, the project gets approved only if enough voters achieve the threshold, and excess signatures play a meaningless role in affecting the results.

Scenario II: If enough contributions are made to reach the stated threshold level of contributions, contributions above the provision point are not wasted, but result in further group benefit and thus more contributions are still meaningful. Herein we assume that the public goods is provided in an amount increasing with the aggregate level of contributions even though the specified threshold has already been met. The evenly distributed benefit is further assumed to be of the linear form *rc*(*N_c_* + *N_i_*)/*N*, where more contributors will provide larger benefits to the group. A large amount of meaningful and visual examples lend support to the above model setting. The more contributions are raised, the higher probability that a project will be successfully constructed. Returning to the earlier example, the neighborhood residents decide to build the public dam by voluntary contributions. The rates of successful provision and observed efficiency of the project are directly and positively related to the amount of contributions. Clearly, an effective dam requires a minimum of contributions to resist the invasion of flood. While if the required threshold is reached, more contributions exceeding the threshold still remain a significant role for a much more effective dam.

However, real-world dilemmas are typically not models with an obvious or clearly defined classification, and thus we combine these two scenarios with a variable *ω* as follows
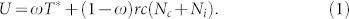
By changing the parameter *ω*, our model allows to transverse smoothly from scenario I (i.e. *ω* = 1) to scenario II (i.e. *ω* = 0). In between the two extremes, we have a mixed situation of these two scenarios in the threshold public goods system.

Further, the payoff to an individual depends only upon her strategy and the combination of the strategies of her opponents. Each player chooses to contribute all or nothing. The proposed TPGG with three strategies has identical allocation rules with the common PGG if the public goods achieves the threshold: each player receives an equal amount of reward from the successful game, minus her own cost related to her strategies. As already stressed, the contributors within a TPGG group are composed by common cooperators (whose number is *N_c_*) and insured cooperators (whose number is *N_i_*). Let us now linger on the game dynamics of the investigated population.

And, we look at the situation the threshold of common goods is attained by
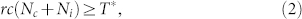
where *r* denotes the amplification effect on the common pool, and T* is the required threshold for the public goods provision to succeed.

Each player derives exclusively from the contributions provided by cooperators and insured cooperators, minus her cost to the common pool. For a group of size *N* probably consisting of the three characters (i.e. cooperator *C*, defector *D* and insured cooperator *I*), the payoffs of these three roles are specified as follows:
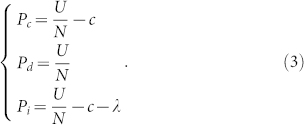
The enhancement factor *r* > 1 means that if all cooperate, they are better off than if all defect. For a public goods game to deserve its name, *r* < *N* should be satisfied, where each individual is better off defecting than cooperating. In this game, each unit of investment is multiplied by *r* and the resulting goods is distributed among all participants irrespective of their strategies. The first term in the expression represents the benefit that the agent obtains from the public goods, while the second term denotes her cost. For a cooperator, the cost is the investment *c* to the public goods. For an insured cooperator, the cost is the contribution *c* to the common pool and her payment *λ* to the insurance. Still, defectors withhold their share and exploit other players.

If the contributions are not sufficient to provide the public goods,
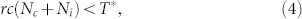
the contributors lose their contributions and the goods is not provided finally. Thus, the net payoffs of the three strategies are determined by
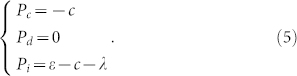
Compared with formula (3), each player is better off if the goods is provided than if it is not. For insured cooperators, they will be compensated by the insurance against the risk of ‘wasting' their contributions on this unrealized project. Thus, the payoff advantages of defectors over insured cooperators depend on the involved parameters: the cooperative contribution *c*, the compensation *ε* (*ε* > 0) provided by the insurance, and the insurance cost *λ*. So, it is difficult to say whether those who do not contribute are better off than those who do contribute.

**Figure 1:**
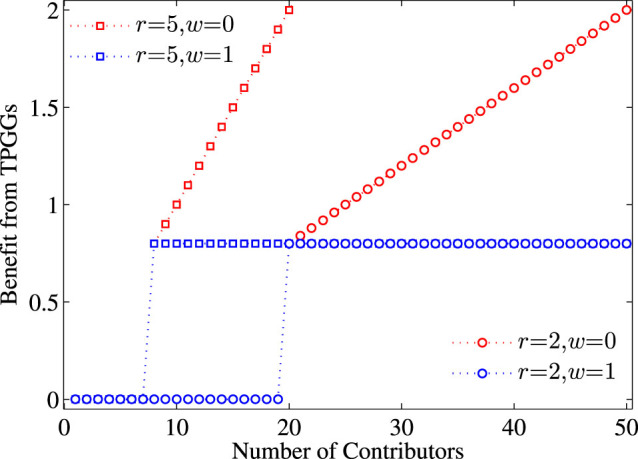
Diagrams illustrating four examples of TPGG, whose dynamics outcomes are closely related to the model parameter involved. Parameters here: *N* = 50, T* = 40. The *x*-axis is indexed by the number of contributors (including cooperators and insured cooperators), and the *y*-axis represents the individual benefits from TPGG. Results show that, when *r* = 5, individuals can gain positive benefits from TPGG if there are at least 8 contributors. When *r* = 2, at least 20 contributors in one TPGG are needed to bring each participant with positive benefits. As mentioned, varying the parameter *ω* can transverse the model smoothly from scenario I (i.e. *ω* = 1) to scenario II (i.e. *ω* = 0) about the payoff functions in the TPGG after the threshold point has already been reached. In between the two extremes, we obtain a mixed situation of the payoff distribution rules in the threshold public goods system.

For simplicity and without loss of generality, we set the cooperative cost *c* from a contributor (either a cooperator or an insured cooperator) to 1. For *r* > 0, we can rewrite *rc*(*N_c_* + *N_i_*) ≥ T* as *N* − *N_d_* ≥ (T*/*r*), and thus introduce *H* = ceil[*N* − (T*/*r*)]. Notably, this ceiling function of *H* returns the smallest integer greater than or equal to *N* − (T*/*r*). Substituting the function *H* for T* thus yields a simple judgment: *N_d_* < *H* leads to the success provision of the TPGG, and *N_d_* ≥ *H* means the failure of the game. In the following study, we employ the threshold value *H* as the maximum number of defectors above which public goods game ends in failure. In this model, the resulting dynamics will be closely related to a variety parameters, as illustrated in Fig. 1 which provides some examples of the proposed TPGG.

## Evolutionary dynamic outcomes

Here we posit a very large, well-mixed population of players. From time to time, sample groups of *N* such players are chosen at random and could join in a threshold public goods game. Notably, the probability that two players in large populations ever encounter again can be neglected. The probability that there are *m* defectors among the *N* − 1 other agents in the sample population of size *N* in which a given player finds herself, is determined by
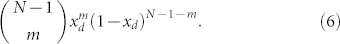
This probability is independent of whether the agent is a contributor or a defector. *x_d_* denotes the fraction of defectors in the population. The only determinant in the well-mixed population is the payoff that the agent herself receives. Consequently, the expected payoff for a defector in such a group is
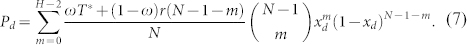
The payoff of a cooperator is given by
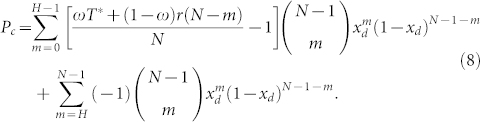
The payoff of an insured cooperator will thus be
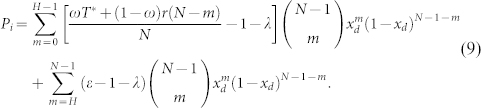


Further, the advantage of one strategy over another depends on the payoff difference between them, below we will discuss the strategy competition results in detail.

Competition between strategy *C* and *I*:
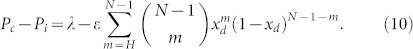
Then we get 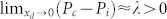, and 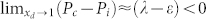. Competition between strategy *I* and *D*:
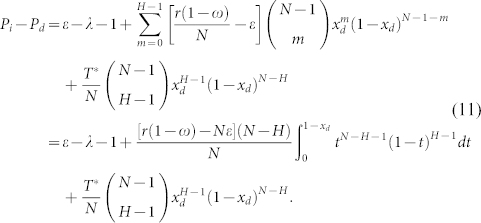


By introducing *ϕ*_1_(*x_d_*), we can rewrite Eq. (11) as
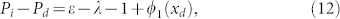
and hence,
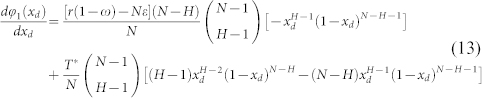


Provided that 0 < *x_d_* < 1 holds, the above Eq. (13) keeps the same sign with −[*r*(1 − *ω*) − *Nε*](*N* − *H*)*x_d_* + T*(*H* − 1)(1 − *x_d_*) − T*(*N* − *H*)*x_d_*. Resolving the equation −[*r*(1 − *ω*) − *Nε*](*N* − *H*)*x_d_* + T*(*H* − 1)(1 − *x_d_*) − T*(*N* − *H*)*x_d_* = 0 yields
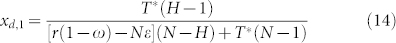


Consequently, both the maximum and minimum values of *ϕ*_1_(*x_d_*) exist, since *ϕ*_1_(*x_d_*) is continuous in [0, 1]. Given that 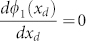 when *x_d_* = *x_d_*_,1_, 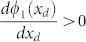 if *x_d_* < *x_d_*_,1_ holds, and 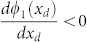 when *x_d_* > *x_d_*_,1_, *P_i_* − *P_d_* reaches the maximum value at *x_d_*_,1_. Then we can safely get 
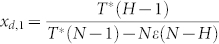
 at *ω* = 1, 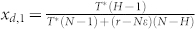 at *ω* = 0.

From Eq. (11) we get 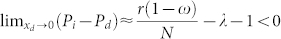, and 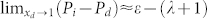.

To sum up, there are two interior roots on the edge of *ID* when *ϕ*_1_(*x_d_*_,1_) + *ε* − *λ* − 1 > 0 and *ε* − (*λ* + 1) < 0, one interior root on the edge of *ID* when *ϕ*_1_(*x_d_*_,1_) + *ε* − *λ* − 1 = 0 and *ε* − (*λ* + 1) < 0 or when *ϕ*_1_(*x_d_*_,1_) + *ε* − *λ* − 1 > 0 and *ε* − (*λ* + 1) > 0, and no interior root on the edge of *ID* when *ϕ*_1_(*x_d_*_,1_) + *ε* − *λ* − 1 < 0.

**Figure 2:**
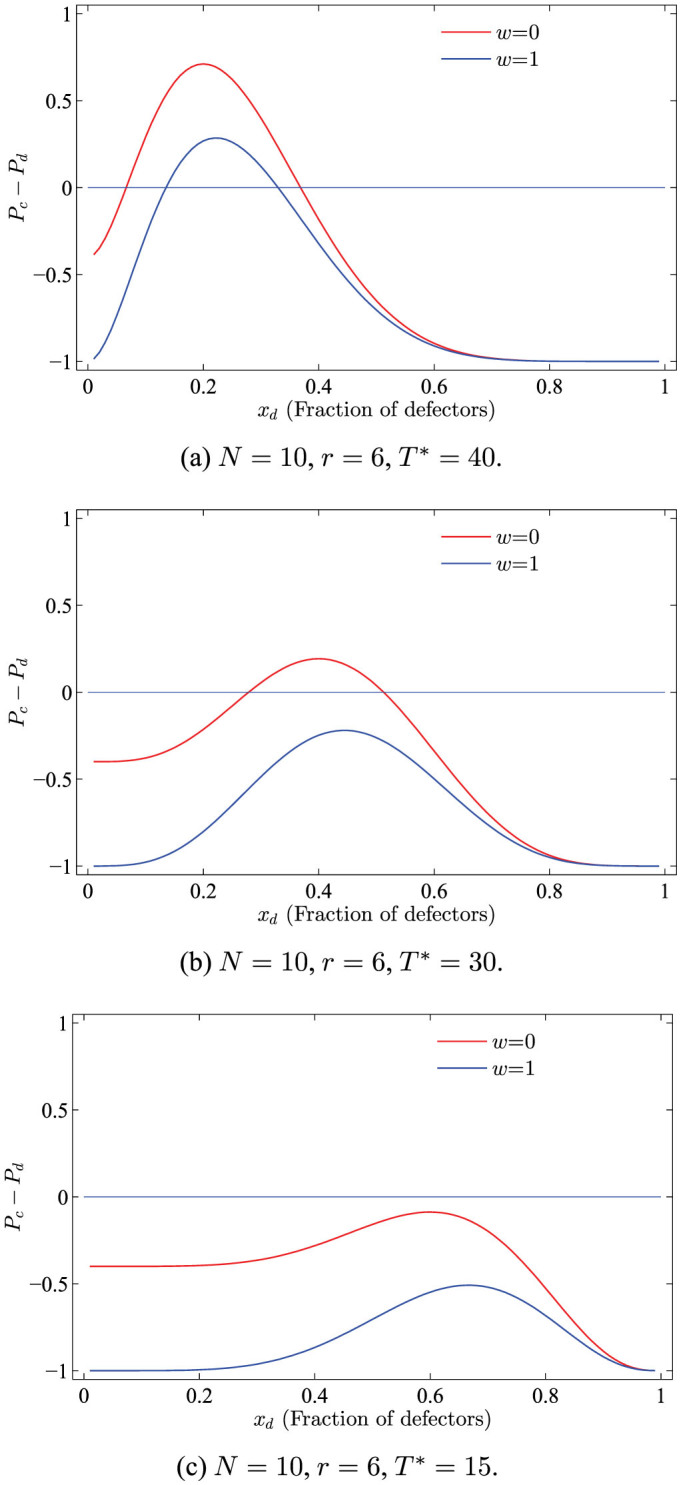
Examples illustrating the payoff difference *P_c_* − *P_d_* between cooperators *P_c_* and defectors *P_d_*, which is closely related to the required threshold T*. Lines connecting the symbols are just to guide the eye. The mentioned examples suggest that the possible roots of the *P_c_* − *P_d_* will be: none, a unique or two roots situated in the interval (0,1).

Competition between strategy *C* and *D*:

In analogy to the above methods, the sign of *P_c_* − *P_d_* determines whether it pays to switch from defection to cooperation or vice versa, with *P_c_* − *P_d_* = 0 being the equilibrium condition. Fig. 2 illustrates three examples with respect to T*, to help depicting the complicated situations of *P_c_* − *P_d_*.
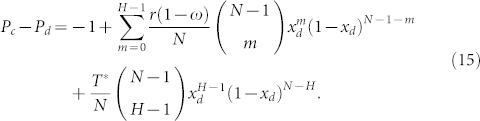
By employing
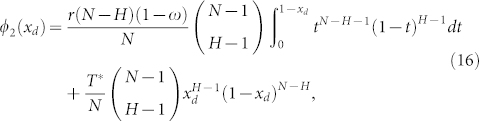
Eq. (15) can be reduced to
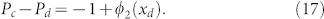
Next,
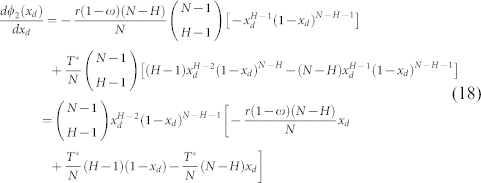


0 < *x_d_* < 1 helps the Eq. (18) keep the same sign with −*r*(1 − *ω*)(*N* − *H*)*x_d_* + T*(*H* − 1)(1 − *x_d_*) − T*(*N* − *H*)*x_d_*. Then,
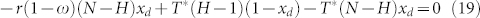
gives rise to
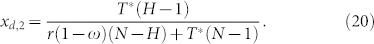


*P_c_* − *P_d_* = −1 when *x_d_* = 0, and *P_c_* − *P_d_* = −1 when *x_d_* = 1. Similarly, *ϕ*_2_(*x_d_*) is a continuous function in the interval of [0, 1], and thus both the maximum and minimum values of *ϕ*_2_(*x_d_*) can be found. Considering that 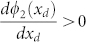 if *x_d_* < *x_d_*_,2_, and 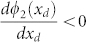 if *x_d_* > *x_d_*_,2_, *P_c_*−*P_d_* reaches its maximum value at *x_d_*_,2_. In this case, *ω* = 1 leads to 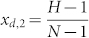, and *ω* = 0 results in 
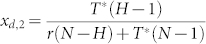
. It thus follows that: there are two interior roots on the edge of *CD* when *ϕ*_2_(*x_d_*_,2_) > 1, one interior root when *ϕ*_2_(*x_d_*_,2_) = 1, and no interior root when *ϕ*_2_(*x_d_*_,2_) < 1.

**Figure 3:**
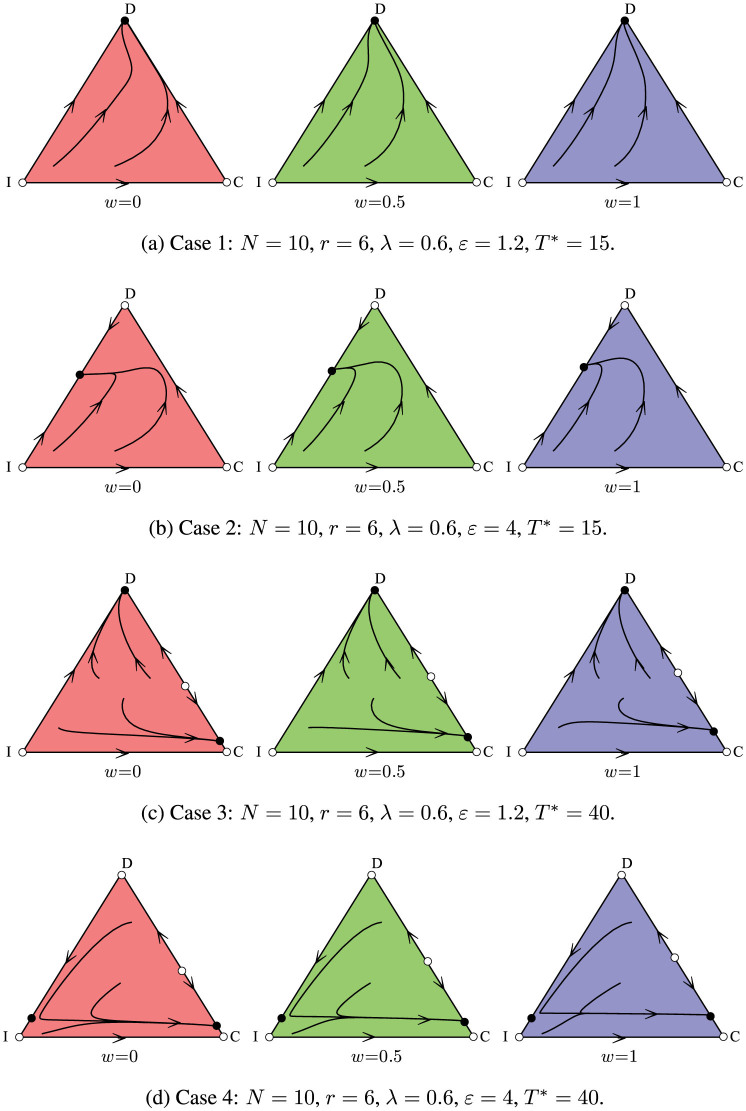
The dynamic outcomes under different cases. The corners *C* (cooperation), *D* (defection), and *I* (insured cooperation) are equilibrium pints. Open dots are unstable equilibrium points and closed dots are stable equilibrium points. In case 1, full *D* is the only stable equilibrium while in the other three cases, other strategies may be the dominative ones. Therefore, we can conclude that our model promotes contribution by adding the third strategy: insured cooperation.

In the continuous time model, the evolution of the fractions of the three strategies are given by
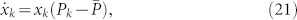
where *k* can be *c*, *d*, *i*, and 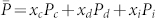. Now consider some typical possible cases of different parameters and the resulting game dynamics one by one, pointed out by Fig. 3.

Case 1 (*ε*−*λ*−1 + *ϕ*_1_(*x_d_*_,1_) < 0, *ϕ*_2_(*x_d_*_,2_)−1 < 0): In this case, full defection equilibrium (*D*) is the only stable and a global attractor. For an insured cooperator, her contribution for common goods and the cost for insurance cannot be totally reimbursed and thus she suffers negative payoffs if the threshold is not reached. Each individual has an incentive to free ride for the higher payoffs, and thus the dominant strategy equilibrium in case 1 is the defection.

Case 2 (*ε* − *λ* − 1 + *ϕ*_1_(*x_d_*_,1_) > 0, *ε* − *λ* − 1 > 0, and *ϕ*_2_(*x_d_*_,2_) − 1 < 0): Herein, there is a border equilibrium consisting of insured cooperation and defection. And this equilibrium is stable and a global attractor. In comparison with case 1, the compensation *ε* from insurance is increased and the resulted *ε* < *λ* + 1 will foster the survival of insured cooperators gaining higher payoffs than defectors. Thenceforth, larger compensation provided by insurance will stimulate more contributors to jointly produce the threshold public goods when they face the ambiguous risks and losses.

Case 3 (*ε* − *λ* − 1 + *ϕ*_1_(*x_d_*_,1_) < 0, *ϕ*_2_(*x_d_*_,2_) − 1 > 0): In this case, there are two border equilibrium points consisting of cooperation and defection. The one close to the full cooperation is a stable equilibrium and the other near full defection is unstable. In comparison with case 1, the increasing threshold T* leads to two stable equilibria here: full defection and the coexistence of cooperation and defection. Which equilibrium the system will evolve to depends on the initial states of the population.

Case 4 (*ε* − *λ* − 1 + *ϕ*_1_(*x_d_*_,1_) > 0, *ϕ*_2_(*x_d_*_,2_) − 1 > 0): In this case, there are two stable border equilibria: one consisting of cooperation and defection, and the other consisting of insured cooperation and defection. In comparison with case 2, the increment of required threshold T* results in the two equilibria on the edge of *CD* here. Similar to case 3, lager *ω* will propel the equilibrium point on the edge of *CD* to approach to the point of pure defection. We offer an accessible explanation of this observation: larger *ω* implies a bigger competitive advantage of defectors over cooperators based on payoffs, which is essential for the stability of the competing strategies.

Summarizing the four cases above, we can conclude that the insurance guarantee for contributive behaviors encourages contributions and provision, but in a manner which interacts with both the required threshold and the reimbursed compensation from insurance. Results presented above show that larger required threshold T* helps contributors gain more advantages in payoffs than free riders, therefore improving the provision for public goods. In addition, increasing the compensation *ε* from insurance also dramatically alters the dynamic outcomes of the game. Defectors reap the benefit of the common goods without any contribution into it, which inhibits the spread of contributive behaviors. Although defectors always do better than cooperators in the public goods games with binary contributions, insurance proposed here can offer the possibility for contributors of receiving higher payoffs than defectors, and so contributors will increase. The insurance reduces or removes the risk that contributions made towards the public goods will be lost if the threshold is not attained. Supported by sufficiently high compensation *ε*, contributors can avoid extinction by the potential payoff advantages over defectors, or even the possible dominance of the population. It is also worth emphasizing that the allocation rules (adjusted by *ω* here) of the public goods after the contributions reach the threshold point, also act as a focal point for survival of cooperation. Smaller *ω* enhances the payoff advantages of contributors over defectors and hence cooperation thrives in our model. Hence, the insurance guarantee encourages contributions and provision in threshold public goods games, and suggests a positive role in unriddling the bewilderment of the ‘Tragedy of the Commons'.

## Conclusions

In the threshold public goods game, public goods are provided if the joint contributions meet or exceed a predetermined threshold level of provisions; otherwise, no public goods is provided. With the existence of the potential risks, we are interested in the capacity of agents to contribute and to produce the public goods when they can opt to be insured at some cost. Therefore individuals joining the game are provided with three strategy options: cooperation, defection and insured cooperation. Here, the public goods is provided in a threshold fashion with a predetermined threshold T*: if the accumulated contributions reach or exceed T* then the public goods is provided, otherwise it is not. In addition, the public goods are allocated according to two different rules if the contributions exceeds the threshold: fixed value or a linear form of contributors. In this model our attention is paid to relating individual contributions in threshold public goods game to riskiness and risk aversion mechanisms.

Theoretical computations show that the evolutionary dynamics are intrinsically regulated by the game parameters specified by the proposed insurance choice. We demonstrate that compensation from insurance is of crucial importance for stabilizing cooperation among competing strategies. Larger compensation will tempt more agents to contribute, thus inhibiting the spread of free riding behavior. Further, increasing the threshold can also elicit more contributions to the threshold public goods game. And, the allocation rules of the public goods after the contributions catch up with the threshold point, also notably affect the final results.

Researchers are often intrigued by employing public goods games to simulate collective dilemmas existing in the real world. In this endeavor, incorporating features of the real-world dilemma into the game also deserves attention. Our work is therefore a potential remedy to collective cooperation problem nested within a dilemma when cooperators are provided with some insurance, implying that the insurance for competing strategies deserves more attention in theoretical and empirical studies. The work reported here also lends itself to multiple extensions. An immediate one, for example, would be improving the theoretical validity of the study here by introducing insurance in experimental research. One feasible experimental research is to conceive of a threshold public goods game with more complicated forms or functions (usually nonlinear) of insurance. Moreover, it would be interesting to see whether insurance provided for both cooperators and defectors in the populations can foster cooperation. For instance, the volunteers in experiments can face multiple actions (e.g., cooperation, defection, insured cooperation and insured defection). In this way, we can gain a thorough understanding about the roles of insurance in the real-life collective dilemmas: such as the construction of some public projects and in other cases where a public good needs to be provided. A closer look at the nature of insurance in situations that are called collective dilemmas can foster the advancement of our understanding of cooperative and selfish behaviors. Hence, learning how insured agents can forgo individual interests for collective interest is useful for understanding social behaviors and developing social policy.

## Author Contributions

J.Z., C.Z. and M.C. performed analyses, discussed the results, and contributed to the text of the manuscript.
